# Functionalized Cellulose Nanocrystal Nanocomposite Membranes with Controlled Interfacial Transport for Improved Reverse Osmosis Performance

**DOI:** 10.3390/nano9010125

**Published:** 2019-01-20

**Authors:** Ethan D. Smith, Keith D. Hendren, James V. Haag, E. Johan Foster, Stephen M. Martin

**Affiliations:** 1 Department of Chemical Engineering, Virginia Tech, Blacksburg, VA 24060, USA; ethands@vt.edu; 2 Macromolecules Innovation Institute, Virginia Tech, Blacksburg, VA 24060, USA; kdh25@vt.edu (K.D.H.); johanf@vt.edu (E.J.F.); 3 Department of Materials Science and Engineering, Virginia Tech, Blacksburg, VA 24060, USA; haagjac@vt.edu; 4 National Center for Earth and Environmental Nanotechnology Infrastructure, Virginia Tech, Blacksburg, VA 24060, USA

**Keywords:** reverse osmosis, thin-film composite, cellulose, nanocomposite, nanocrystal

## Abstract

Thin-film nanocomposite membranes (TFNs) are a recent class of materials that use nanoparticles to provide improvements over traditional thin-film composite (TFC) reverse osmosis membranes by addressing various design challenges, e.g., low flux for brackish water sources, biofouling, etc. In this study, TFNs were produced using as-received cellulose nanocrystals (CNCs) and 2,2,6,6-Tetramethylpiperidine-1-oxyl (TEMPO)-oxidized cellulose nanocrystals (TOCNs) as nanoparticle additives. Cellulose nanocrystals are broadly interesting due to their high aspect ratios, low cost, sustainability, and potential for surface modification. Two methods of membrane fabrication were used in order to study the effects of nanoparticle dispersion on membrane flux and salt rejection: a vacuum filtration method and a monomer dispersion method. In both cases, various quantities of CNCs and TOCNs were incorporated into a polyamide TFC membrane via in-situ interfacial polymerization. The flux and rejection performance of the resulting membranes was evaluated, and the membranes were characterized via attenuated total reflectance Fourier transform infrared spectroscopy (ATR-FTIR), transmission electron microscopy (TEM), and atomic force microscopy (AFM). The vacuum filtration method resulted in inconsistent TFN formation with poor nanocrystal dispersion in the polymer. In contrast, the dispersion method resulted in more consistent TFN formation with improvements in both water flux and salt rejection observed. The best improvement was obtained via the monomer dispersion method at 0.5 wt% TOCN loading resulting in a 260% increase in water flux and an increase in salt rejection to 98.98 ± 0.41% compared to 97.53 ± 0.31% for the plain polyamide membrane. The increased flux is attributed to the formation of nanochannels at the interface between the high aspect ratio nanocrystals and the polyamide matrix. These nanochannels serve as rapid transport pathways through the membrane, and can be used to tune selectivity via control of particle/polymer interactions.

## 1. Introduction

Access to clean drinking water is one of the foremost challenges faced by engineers and scientists today. Approximately one-third of the world’s population lacks access to clean water, and by 2025, it is expected that this statistic will increase to two-thirds [1]. Desalination technologies have been heavily researched as a solution to provide clean water, given that 96.5% of the world’s water is located in seas and oceans [2]. Membrane-based reverse osmosis (RO) processes are currently the largest method of desalination, accounting for 63.7% of all desalination methods and a global capacity of around 100 million m^3^ per day [3]. Much of the desalination research over the past 10 years has focused on improving the efficiency of membrane technologies in order to lower the production cost for large-scale processes [4].

The majority of current RO processes use aromatic polyamide (PA)-based thin-film composite (TFC) membranes based on a design developed in the late 1970s by Cadotte [5]. Thin-film composite membranes consist of several layers: a support layer generally made of non-woven polyester, a porous polysulfone layer, and a selective aromatic polyamide layer. Thin-film composite membranes possess several advantages over other polymeric membranes, such as cellulose acetate membranes, including the ability to fine tune the properties of each layer, stability over large pH and temperature ranges, lower operating pressure requirements, and higher flux and rejection of salts and organics [6,7]. Therefore, TFCs have been the subject of many recent studies which have sought to modify existing membrane designs to improve their energy efficiency and address issues such as surface fouling and resistance to chemical attack [8]. Adding nanoparticles to TFC membranes results in a new class of thin-film nanocomposite (TFN) membranes. In this context, nanoparticles are of interest due to their unique morphologies, surface chemistry, pore structures, and their ability to influence crosslinking during interfacial polymerization (IP) [9]. Membrane performance studies have been conducted using nanoparticles such as our prior work using single-walled carbon nanotubes (SWNTs) [8], and the work of other groups using NaA zeolites [10,11,12], Ag nanoparticles [13], silica [14], metal organic frameworks (MOFs) [9,15,16], and TiO_2_ [17,18,19]. A summary of the results of some of these studies is given in Table A1 in the Appendix A. Due to the high cost of many nanoparticles, such as carbon nanotubes, this work focuses on cellulose nanocrystals (CNCs) as a cost-effective alternative.

**Figure 1:**
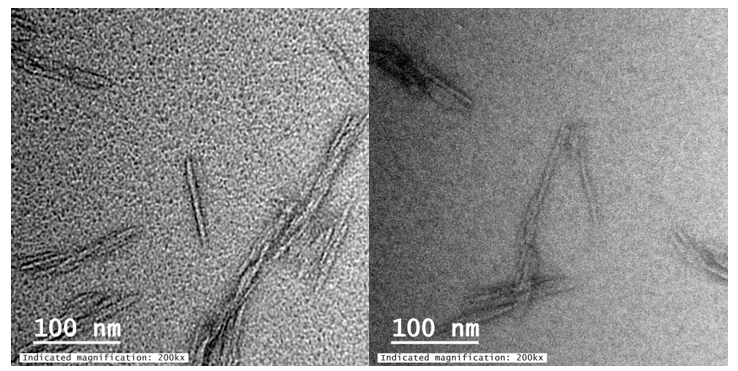
Transmission electron microscopy (TEM) micrographs of 2,2,6,6-Tetramethylpiperidine-1-oxyl (TEMPO)-oxidized cellulose nanocrystals (TOCNs) used in this work. Imaging techniques are described in Section 2.3.3.

Cellulose nanocrystals (CNCs) are high aspect ratio nanoparticles produced via the acid hydrolysis of cellulose nanofibers. Cellulose nanocrystals are desirable as a potential modifier for TFN membranes due to their low cost, sustainability, attractive mechanical properties, and potential for surface modification [20]. The high aspect ratio of CNCs is of particular interest due to our previous work with carbon nanotubes, which possess similar dimensions [21]. The potential for functionalization is an important attribute since certain functional groups on other nanoparticles have shown improved flux, rejection, and diminished potential for biofouling [8]. Following sulfuric acid hydrolysis, cellulose nanocrystals possess charged surface sulfate groups that result from the esterification of surface hydroxyl groups [22]. Many surface modification techniques exist to change these surface functionalities in order to alter the properties of the nanocrystals [20,23,24] such as 2,2,6,6-Tetramethylpiperidine-1-oxyl (TEMPO)-mediated oxidation [20]. TEMPO-oxidized cellulose nanocrystals (TOCNs) have also been previously used as additives in ultrafiltration membranes, showing improved water flux due to increased membrane hydrophilicity and anti-fouling properties [25]. Presently, the literature lacks studies on CNCs as an additive to TFC RO membranes. Cellulose nanocrystals exhibit many different length scales based on their source material [26,27]. Figure 1 exhibits transmission electron microscopy (TEM) micrographs showing the length scale of TOCNs used in this study.

**Figure 2:**
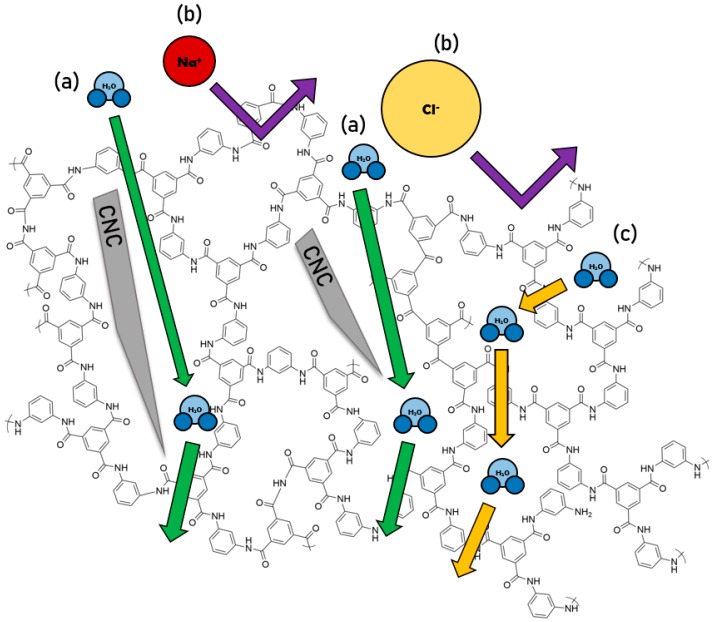
Illustration depicting known and proposed transport mechanisms within a thin-film nanocomposite membrane. (**a**) Improved transport of water molecules via interfacial nanochannel formation due to cellulose nanocrystal (CNC)-polyamide interactions; (**b**) Na^+^ and Cl^−^ ions rejected by polymer matrix; (**c**) conventional water transport through polyamide matrix; diffusion through network voids. Note: depictions of ions and nanoparticles are not to scale.

The majority of TFN membrane fabrication methods involve in situ deposition of nanoparticles, i.e., an interfacial polymerization is performed above or around deposited nanoparticles, as depicted in Figure 2. The nanoparticles interrupt bond formation between monomer and crosslinking agents, leading to the formation of voids in the resulting polymer matrix. In a traditional TFC RO membrane, water molecules diffuse through the polymer matrix as shown by pathway (c) in Figure 2. These network voids are large enough to allow the passage of water molecules, but small enough to reject larger sodium and chlorine ions (b). Nanoparticle addition influences the formation of the polyamide, which introduces alternate transport pathways for water molecules. We hypothesize that high aspect ratio nanoparticles may cause the formation of nanochannels, which are nano-scale voids present down the length of included nanoparticles [21]. This is illustrated for a cellulose nanocrystal by pathway (a). Water molecules can travel more rapidly through nanochannels, causing an increase in water permeance, while maintaining high rejection as long as the nanochannels remain small enough to exclude salt ions.

Previous work in our group has shown that carbon nanotubes can be deposited in a semi-aligned manner on a polyethersulfone (PES) ultrafiltration membrane support layer, which allows the nanoparticles to penetrate into the polyamide during IP [8]. A vacuum filtration apparatus was used to introduce extensional flow, which has been shown to cause alignment of carbon nanotubes in the direction of the flow [28]. While the chemical structures of carbon nanotubes and various types of nanocellulose differ greatly, the high-aspect ratios of cellulose nanocrystals could influence the performance of existing TFC membrane designs in a similar way when dispersed throughout the polyamide thin film. Cellulose nanocrystals have been shown to orient in the direction of flow under specific conditions [29,30], so it is possible that some degree of alignment could be achieved via the vacuum filtration method previously demonstrated by our laboratory for CNT-based TFN membranes. However, a more commonly used method of nanoparticle dispersion involves the addition of the nanoparticles to one of the monomer solutions used in the polyamide interfacial polymerization [31,32,33].

Herein we report on the incorporation of as-received hydroxyl functionalized cellulose nanocrystals (CNCs) and TEMPO-oxidized cellulose nanocrystals (TOCNs) during interfacial polymerization to form thin-film nanocomposite RO membranes. The CNCs and TOCNs were incorporated into membranes via both a vacuum filtration method and a monomer dispersion method. The membrane performance was examined in order to evaluate the effectiveness of CNCs and TOCNs as TFN additives, as well as to draw conclusions about interactions between the particles and the polymer matrix. We hypothesized that for both fabrication methods, the addition of CNCs or TOCNs would lead to increases in water flux due to the formation of nanochannels at the nanocrystal/polymer interface, and that salt rejection performance would depend on the nature of the interactions between the polyamide matrix and the nanocrystal surface.

## 2. Materials and Methods

The following chemicals were purchased from Sigma–Aldrich (St. Louis, MO, USA): 1,3,5 benzene tricarbonyl trichloride (trimesoyl chloride, TMC), m-phenylenediamine (MPD), sodium dodecylbenzenesulfonate (SDBS), sodium bromide (>99%), American Chemical Society (ACS) reagent grade sodium hydroxide (≥97.0%), solution reagent grade sodium hypochlorite (10–15%), ethanol (200 proof), and (2,2,6,6-Tetramethylpiperidin-1-yl)oxyl (TEMPO) free radical (98%). Hexanes, sodium chloride, and dialysis tubing were purchased from Fisher Scientific (Hampton, NH, USA). Polyethersulfone (PES) ultrafiltration membranes were provided by DOW Filmtec (Minneapolis, MN, USA). Cellulose nanocrystals were obtained from the University of Maine (Orono, ME, USA).

### 2.1. Preparation of Nanocellulose

It should be noted that a small amount of sulfate half esters were present on the surface of the as-received CNCs, and were measured previously as approximately 100 mmol/kg [34].

#### 2.1.1. TEMPO Oxidation and Characterization of CNCs

The TEMPO-mediated oxidation was performed on as-received CNCs using a well-known method [35], generating a 5.5 ± 0.3 mg/mL TOCN solution in deionized (DI) water. The TOCN dimensions were determined via TEM imaging (described in Section 2.3.3) and Fiji image processing software [36] to be 101 ± 8 nm by 7 ± 1 nm. Carboxylic acid end group concentration was determined to be 1.16 mmol/g cellulose via conductometric titrations and agreed with reported values [37,38,39]. Further details on titration procedure may be found in Appendix A.

#### 2.1.2. Nanocellulose Stock Solution Preparation

As-received cellulose nanocrystals (11.8 wt% slurry in water) were used to prepare a 10 mg/mL stock solution of CNCs in DI water as well as a 1.0 mg/mL stock solution. The 5.5 mg/mL solution of TOCNs prepared in Section 2.1.1. was further diluted to create a 1.0 mg/mL stock solution of TOCNs in DI water. Both 1.0 mg/mL solutions were used to control the nanocrystal loading in the vacuum filtration method (Section 2.2.2), while the 10 mg/mL and 5.5 mg/mL solutions were used for the dispersion method (Section 2.2.3).

### 2.2. Membrane Fabrication

The interfacial polymerization of polyamide is sensitive to reaction conditions, and methods reporting various monomer concentrations, temperatures, curing times, etc. can be found in the TFN literature [5,8,11,40,41]. Two fabrication methods were explored during this study: a process in which CNCs are added to the membrane via a vacuum filtration step; and a process in which nanoparticles are dispersed within the aqueous monomer solution.

#### 2.2.1. Support Pretreatment

The PES ultrafiltration supports were soaked in a solution of 0.5 wt% SDBS for at least 24 h before being subjected to IP in the vacuum filtration and dispersion methods.

#### 2.2.2. Vacuum Filtration Fabrication Method

**Figure 3:**
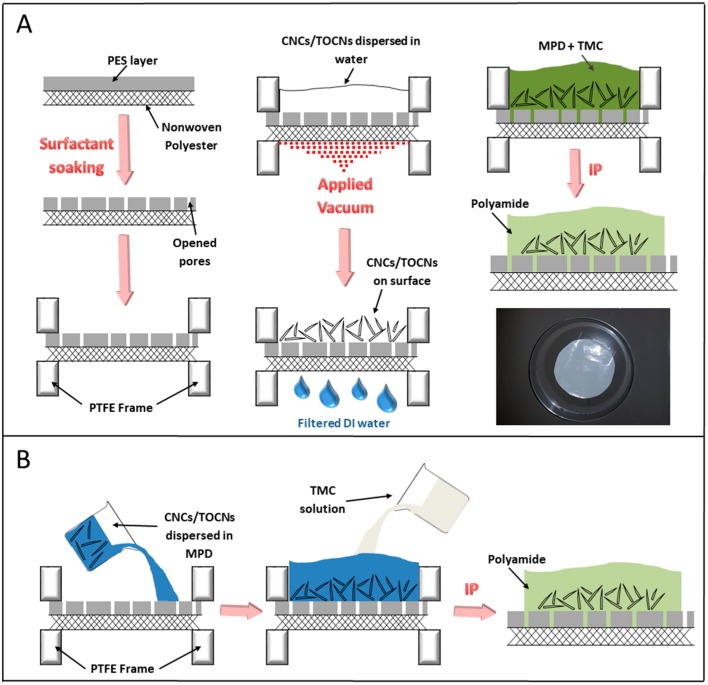
The CNC/TOCN nanocomposite membrane fabrication processes. (**A**) Vacuum filtration method; (**B**) monomer dispersion method. Adapted with permission from W. Chan, H. Chen, A. Surapathi, M. Taylor, Zwitterion Functionalized Carbon Nanotube/Polyamide Nanocomposite Membranes for Water Desalination, ACS Nano. 7 (2013) 5308–5319. Copyright 2013 American Chemical Society.

The vacuum filtration fabrication method is similar to a process previously reported by our group [8] which consists of two steps: deposition of nanocrystals onto a porous support followed by interfacial polymerization of the polyamide, as depicted in Figure 3A. Quantities of the stock solutions generated in Section 2.1.1. corresponding to the desired loading of CNCs and TOCNs were dispersed in a 0.2 wt% SDBS solution via ultrasonication. The pretreated PES support was placed in a vacuum filtration system, and the nanocrystal solutions were poured directly on top. A vacuum was applied to draw the water through the PES support and deposit the nanocrystals onto the surface of the support. Any orientation of the nanocrystals occurs during this step, influenced by the hydrostatic forces of the water passing through the PES support as well as the hydrogen bonding and van der Waals interactions between nanocrystals and with the neutral PES surface [42]. Following vacuum filtration, the support on which the nanocrystals were deposited was placed into a circular polytetrafluoroethylene (PTFE) frame, and submerged in a 2 wt% solution of MPD for 30 min. The support was then removed from the solution and placed on a flat surface. A glass roller was used to remove the excess MPD solution. The support was then placed back into the PTFE frame, where it was submerged in a 0.1 *w*/*v*% TMC solution for 90 s. The resulting aromatic polyamide TFN membrane was then heated for 10 min at 68 °C. After cooling, the membrane was washed and stored in DI water.

#### 2.2.3. Monomer Dispersion Fabrication Method

The CNCs and TOCNs readily disperse in water, therefore 2 wt% MPD aqueous solutions (without added SDBS surfactant) were prepared containing CNC and TOCN concentrations varying from 0.05 wt% to 0.5 wt% using the stock solutions from Section 2.1.2. The PES supports pretreated in a 0.5% SDBS solution were dried and placed into a PTFE frame. The PES supports were submerged in the TOCN/CNC-containing 2 wt% MPD solutions for 30 min, as depicted in Figure 3B. The support was then removed from the solution and placed on a flat surface. A glass roller was used to remove the excess MPD solution. The support was then placed back into the PTFE frame, where it was submerged in a 0.1 *w*/*v*% TMC solution for 90 s. The resulting aromatic polyamide TFN membrane was then heated for 10 min at 68 °C. After cooling, the membrane was washed and stored in DI water.

### 2.3. Characterization

#### 2.3.1. ATR-FTIR

Attenuated total reflection Fourier transform infrared spectroscopy (ATR-FTIR) scans of the polyethersulfone support material, polyethersulfone support material with deposited CNCs, and the final TFN polyamide membrane were performed (Nicolet iS50, ThermoFisher Scientific, Waltham, MA, USA) in air in the mid-infrared region (500–4000 cm^−1^) and 128 scans were averaged at each collection point. An ATR correction was performed on each scan using analysis software (OMNIC™, ThermoFisher Scientific, Waltham, MA, USA). Data were plotted in Microsoft Excel. No additional data manipulation was performed.

#### 2.3.2. AFM Imaging and Surface Roughness

Surface images (height and phase contrast) and roughness values were obtained using atomic force microscopy (AFM) (Asylum Research Cypher ES, Santa Barbara, CA, USA). Tapping mode scans were performed at various scales using Oxford Instruments AC160TS-R3 probes (k = 26, f = 300 kHz). Root mean square roughness values were obtained using AFM analysis software (Igor Pro, Asylum Research, Santa Barbara, CA, USA) on at least three scans from identical membranes.

#### 2.3.3. TEM

Transmission electron microscopy (TEM) was used to characterize the sizes of TOCNs used in this work. Images were acquired using a JEOL 2100 Transmission Electron Microscope (JEOL, Ltd., Peabody, MA, USA) and previously described imaging techniques [43].

Transmission electron microscopy was also conducted in order to directly visualize the membrane layers of interest. Specimens were prepared by cutting radial slices from the interior of the film samples and set in Epo-Fix epoxy resin to cure for 24+ h. An RMC Powertome PC (Boeckeler Instruments, Tucson, AZ, USA) microtome was used at room temperature to collect electron transparent sections on 3 mm hexagonal mesh copper grids.

Analysis was performed on a thermionic Philips EM420 transmission electron microscope at an acceleration voltage of 120 kV. Images were acquired in bright-field conditions and recorded on a charge-coupled device (CCD) camera. Fiji [36] was used to process the images and measure the thicknesses of the polyamide skin layers.

### 2.4. Membrane Permeation Evaluation

Salt rejection and flux data were determined using a reverse osmosis testing system (Sterlitech Corporation, Kent, WA, USA). The TFN membranes fabricated via interfacial polymerization were masked between two layers of aluminum tape (VentureTape, 3M, Minneapolis, MN, USA) sealed with epoxy (The Gorilla Glue Company, Cincinnati, OH, USA) and allowed to cure for 24 h. Photographs of each membrane were taken, and the active area of the membrane was measured via Fiji (average membrane active area was 5.55 ± 0.25 cm^2^). Masked membranes were placed into the 316 Stainless Steel test cell and subjected to a 2000 ppm Na^+^ feed solution at a flow rate of 2.5 LPM and pressure of 250 psi. A cooling system was used to maintain a constant feed temperature of 20 ± 2 °C. Permeate samples were collected over a set period of time and weighed. Flux values for each membrane were calculated via Equation (1):

(1) $$Q=\frac{\Delta V}{A\cdot t},$$

where Q is flux, ΔV is the volume of the permeate obtained by dividing the mass of each permeate sample by the density of each sample (low salt concentrations were assumed, therefore the density was approximately the density of water, 0.997 g/cm^3^), A is the active area of each membrane, and t is the time over which the sample was collected.

Sodium ion concentrations in the feed solution and permeate samples were obtained via atomic absorption spectrophotometry (Buck Scientific, Norwalk, CT, USA) and used in conjunction with Equation (2) to calculate rejection as a percentage.

(2) $$ℜ(\%)=\left(1-\frac{C_{p}}{C_{f}}\right)\times 100,$$

where ℜ is rejection (as a percentage), *C_p_* is the concentration of cations in the permeate, and *C_f_* is the concentration of cations in the feed.

## 3. Results

### 3.1. ATR-FTIR

**Figure 4:**
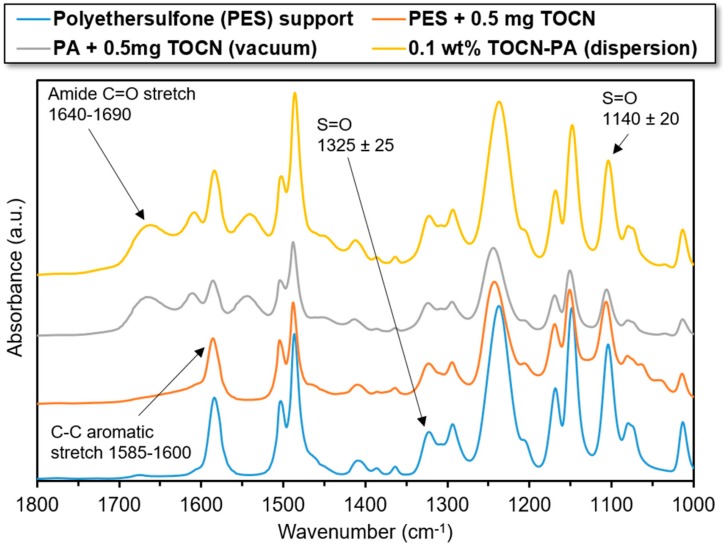
FT-IR scans (wave numbers 1000–1800 cm^−1^) of polyethersulfone (PES) support material, PES with 0.5 mg TOCNs deposited, and thin film nanocomposite (TFN) polyamide membrane with 0.5 mg TOCN loading.

**Figure 5:**
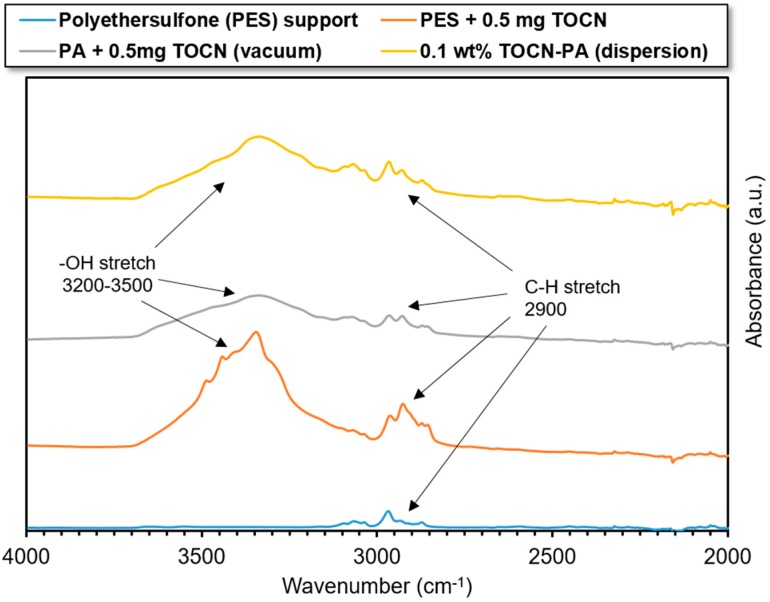
FT-IR scans (wavenumbers 2000–4000 cm^−1^) of PES support material, PES with 0.5 mg TOCNs deposited, TFN polyamide membrane with 0.5 mg TOCN loading (vacuum filtration fabrication), and 0.1 wt% TOCN TFN membrane (dispersion fabrication method).

Attenuated total reflectance (ATR) spectroscopy has a scan depth of approximately 0.5–2.0 µm, depending on the refractive indices of the sample and ATR crystal and the scan wavenumber [44]. Given that the polyamide skin layers of TFN membranes are generally 200–400 nm in thickness, ATR spectroscopy is an ideal technique for determining the composition of the surface layers. The ATR-FTIR scans were performed on a PES support membrane before and after vacuum filtration of 0.5 mg TOCNs as well as on TFN membranes, one made with the vacuum filtration fabrication method (0.5 mg TOCN) and dispersion fabrication method (0.1 wt% TOCN). The results of these scans are shown in Figure 4 and Figure 5.

Figure 4 shows characteristic peaks of polyethersulfone at wave numbers 1140 cm^−1^, 1325 cm^−1^, and in the range 1585–1600 cm^−1^. These peaks remain strongly present in the scans with deposited TOCNs and after interfacial polymerization via both methods. The characteristic amide C=O peak appears following interfacial polymerization in the region 1640–1690 cm^−1^ for the scans from TFN membranes. The polysulfone characteristic peaks (1140 and 1325 cm^−1^) appear to diminish following TOCN deposition via vacuum filtration. This implies that the TOCNs are completely covering the support in the scanned region. More evidence for this conclusion is provided in Section 3.3. Furthermore, the polyethersulfone characteristic peaks are stronger in the scan for the 0.1 wt% TOCN membrane. This evidences that the TOCNs are more well-distributed throughout the skin layer of the TFN. This is further shown in Figure 5, which shows the 2000–4000 cm^−1^ region of ATR-FTIR scans.

Figure 5 confirms the presence of TOCNs, where peaks for both surface hydroxyl groups (3200–3500 cm^−1^ stretch) and additional signal strength for the C–H stretch (2900 cm^−1^) may be observed in all samples except the pristine PES support. For the vacuum filtration fabrication method, these peaks strengthen after TOCN deposition on the PES support material and drop in intensity after interfacial polymerization, indicating that the TOCNs have been completely covered by the polyamide skin layer. A diminished –OH stretch peak may also be observed in the 0.1 wt% TOCN scan; however, the peak is stronger in intensity than the peak for the vacuum filtration fabrication method. The stronger signal is likely due to better dispersion of TOCNs throughout the polyamide skin layer. It is important to note that the 0.1 wt% TOCN membrane was chosen for this comparison since it is the second-lowest loading of TOCNs used in the dispersion method, and therefore demonstrates that even at low loading via the dispersion method, the signal from the nanoparticles is stronger than the comparable signal from the vacuum filtration scans.

### 3.2. TEM

#### 3.2.1. Size of TEMPO-Oxidized Cellulose Nanocrystals

Transmission electron microscopy was used to characterize the size of TOCNs used in this work. The TEM micrographs in Figure 1 were obtained and processed, and the average dimensions of the TOCNs were determined to be 101 ± 8 nm by 7 ± 1 nm.

#### 3.2.2. Membrane Cross-Sections

**Figure 6:**
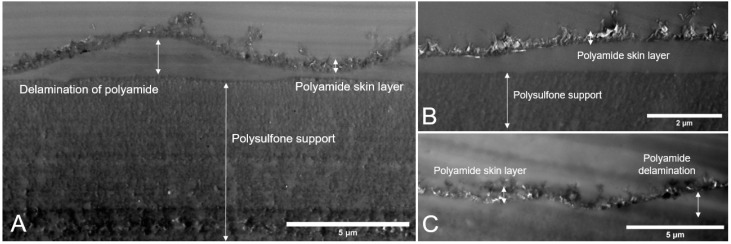
Cross-sectional TEM images of membranes fabricated via the vacuum filtration method: (**A**) plain polyamide TFC membrane; (**B**) TFN membrane with 0.5 mg of CNCs; and (**C**) and TFN membrane with 0.5 mg TOCNs. Delamination of the polyamide layer may be observed in all three samples, likely due to sample preparation conditions. Images were used to obtain thickness values for the polyamide layer in each case.

Transmission electron microscopy is often used to characterize the thickness of TFC polyamide skin layers. In TFN studies, TEM images may give valuable insight into the dispersion of nanoparticle additives [13,31,32]. Therefore, TEM images of the cross-section of the membranes were obtained for TFN membranes made via the vacuum filtration method. Figure 6 shows the polyamide skin layer formed via IP for three cases. Figure 6A depicts the cross section of the plain polyamide TFC membrane, while the TFN membranes with 0.5 mg of CNCs and TOCNs (fabricated via vacuum filtration method) are depicted in Figure 6B,C respectively. The polyamide skin layers and polyethersulfone support layers are clearly defined.

It should be noted that delamination of the polyamide layer is observed for all membrane samples and likely occurred during TEM sample preparation. Generally, the polyamide skin layer is held against the polysulfone support by van der Waals forces resulting from polymer formation within the pores of the support; however, it has been posited that the polyamide layer may form a small quantity of covalent bonds under certain reaction conditions via a Friedel–Crafts acylation mechanism [45]. The strength of this adhesion may be overcome by the curing process of the epoxy, resulting in the delamination of the polyamide layer seen in all three images in Figure 6. The presence of the delamination in the control sample (Figure 6A) provides evidence that polyamide delamination is not a consequence of the addition of nanocellulose to the membrane.

The TEM images do not confirm the presence of cellulose nanocrystals in the polyamide skin layer or provide insight into their dispersion. This is due to the lack of contrast between the nanocrystals and the polyamide present in the system [34]. Based on these results, TEM imaging was not performed on membranes fabricated via the dispersion method, as we believe no useful insight on TOCN or CNC dispersion would be gathered. However, the images in Figure 6 were used to characterize the polyamide layer thickness.

Imaging software was used to analyze the layer thickness using the cross-sectional TEM images for each membrane, the results of which are presented in Table 1. Polyamide layer thickness does not vary significantly between membranes. A slight decrease (42 nm) in the average thickness is noted for the 0.5 mg CNC membrane; however, it is likely that this decrease occurred due to slight differences in the interfacial polymerization conditions during sample preparation.

### 3.3. Atomic Force Microscopy (AFM)

**Figure 7:**
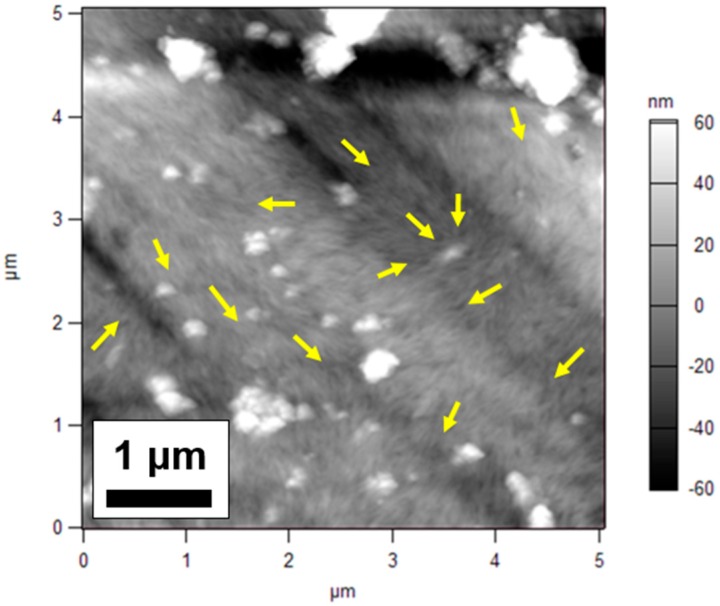
Atomic force microscope (AFM) height retrace image of PES support with 0.5 mg TOCNs deposited on the surface. Arrows indicate various directions of orientation of TOCNs.

Unlike electron microscopy techniques, atomic force microscopy (AFM) relies on a physical cantilever to scan samples, making AFM a more useful technique when characterizing polymeric systems with nanocellulose [34]. Nanoparticle orientation within TFN membranes is of key importance given that CNCs have been shown to exhibit ordering when exposed to shear forces [46] and may organize into chiral nematic liquid crystals above certain particle concentrations [47]. The AFM scans were performed on the surfaces of PES supports following vacuum filtration of nanocellulose solutions. Large aggregations are visible at scan sizes above 5 µm at all loading levels. Figure 7 shows an AFM height retrace scan of TOCNs deposited on a PES support at 0.5 mg loading.

The yellow arrows in Figure 7 indicate various orientation directions within the plane of the PES support surface. This suggests that when subjected to forces during vacuum filtration, the TOCNs did not exhibit alignment in a vertical direction, but rather compacted into a solid layer aligned in the plane of the membrane as the water was removed from the system. Furthermore, the concentration of TOCNs at 0.5 mg loading resulted in many aggregated clumps of TOCNs across the surface of the membrane. Both of these effects are common challenges associated with characterizing CNC systems that involve drying out suspensions of the particles [34]. The AFM scans confirmed that the orientation of CNCs and the shapes and sizes of CNC aggregates varied significantly from membrane to membrane.

**Figure 8:**
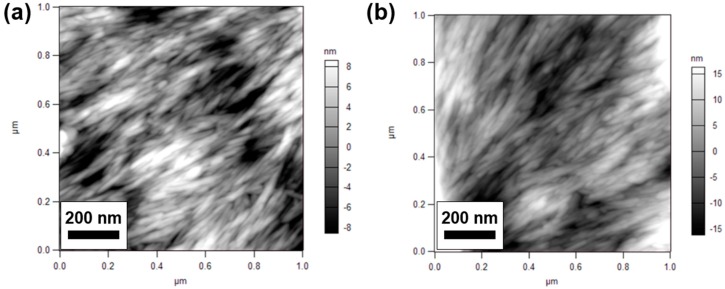
AFM height retrace images of (**a**) 0.5 mg TOCN loading and (**b**) 0.75 TOCN loading at the one-micron scan size (phase contrast images over the same region are included in Appendix A).

The AFM scans also indicated that TOCNs were packed tightly across the surface. For TOCNs, this effect is enhanced by the hydrogen bonding interactions between the carboxyl surface groups, but is primarily due to strong van der Waals interactions between the nanocrystals [48] and the aforementioned tendency of CNCs to orient when drying. Figure 8 shows 1 µm AFM scans of PES supports with either (a) 0.5 mg or (b) 0.75 mg of TOCNs deposited during the vacuum filtration fabrication method. The packing of the nanocrystals is evident in both scans and is similar for both loadings. The root mean square (RMS) roughness values were higher for the 0.75 mg loading (8.630 nm) than for the 0.5 mg loading (3.938 nm), but this is largely attributable to the presence of TOCN aggregates, which can be seen in the top right and lower left of Figure 8b.

The dense CNC layers formed on the surface of the PES supports are not ideal for the production of TFN membranes. The CNCs may form a barrier layer that blocks the pores of the PES support leading to a decrease in water pathways through the membrane. Large aggregates may also cause defects in the polymer matrix leading to decreased selectivities in the TFC membranes. Furthermore, a solid layer of nanocellulose following vacuum filtration does not promote effective dispersion of nanoparticles during the subsequent interfacial polymerization that would lead to improved membrane performance.

**Figure 9:**
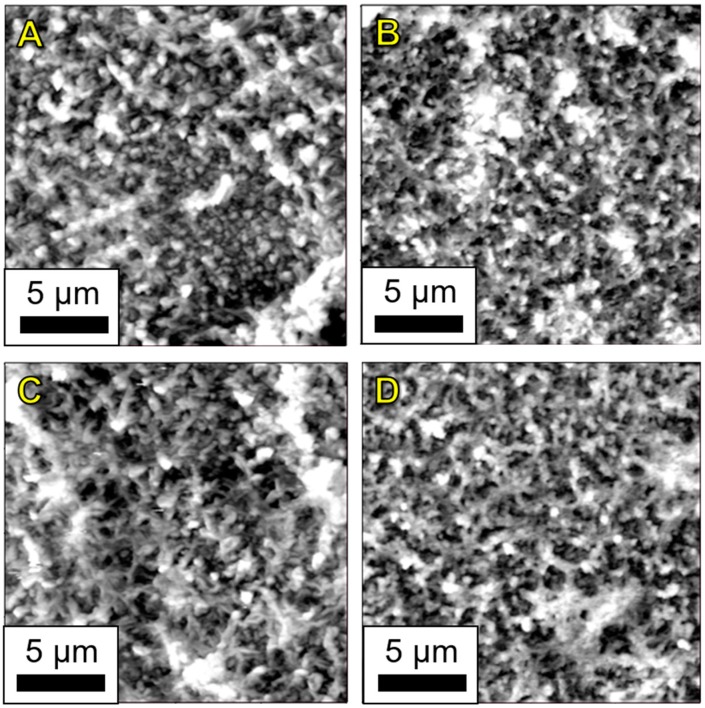
AFM height retrace scans of (**A**) plain polyamide, (**B**) 0.75 mg TOCN, vacuum filtration method, (**C**) 0.5 wt% TOCN, dispersion method, and (**D**) 0.5 wt% CNC dispersion method.

**Figure 10:**
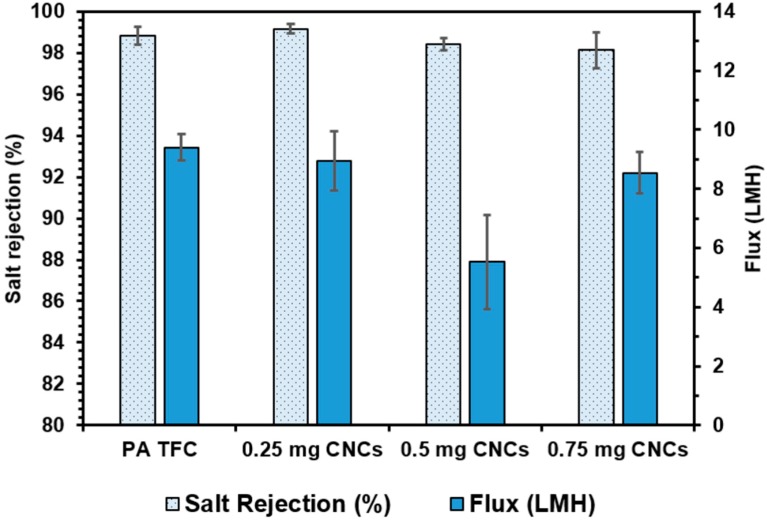
Flux and salt rejection results for CNC containing TFN membranes fabricated using the vacuum filtration method.

The AFM imaging was conducted on membranes fabricated via the vacuum filtration and dispersion methods to determine if the addition of nanoparticles impacted the polyamide skin layer structure and surface roughness. Figure 9 shows AFM height retrace scans of membranes fabricated with both methods. All of the images were consistent with the expected structure of interfacially polymerized polyamide reported across the membrane literature [32,33,49,50]. No distinct structures were observed that appear to be cellulose nanocrystals or aggregates of crystals, even at the highest loading levels (Figure 9B–D). In the case of the vacuum filtration method, the polyamide skin layer entirely covers the deposited layer of TOCNs. For the dispersion method, it is likely that the nanocrystals are well-dispersed through the polyamide layer at all loading levels. The RMS roughness data were calculated from images at each loading level. The polyamide control membrane was determined to have an RMS roughness of 180 ± 20 nm, the 0.75 mg TOCN vacuum filtered membrane 212 ± 3 nm, the 0.5 wt% TOCN dispersion membrane 130 ± 19 nm, and the 0.5 wt% CNC membrane 158 ± 8 nm. For the membranes displayed in Figure 10, roughness decreased for the membranes fabricated via the dispersion method, while the roughness increased for the vacuum filtered membrane. The presence of CNCs may have affected the surface formation of the polymer, but it is more likely that these variations are due to small changes in interfacial polymerization reaction conditions or the choice of location for RMS measurements. Further details for the RMS measurements are given in Appendix A.

### 3.4. TFN Membrane Performance

#### 3.4.1. Vacuum Filtration Fabrication Method

Figure 10 shows the RO performance data for TFNs modified with CNCs and fabricated via the vacuum filtration method. The presence of the CNCs did not significantly impact the rejection properties of these membranes at any loading level. The CNCs were used as-received from the manufacturer, and the majority of the surface functional groups are hydroxyl or hydroxymethyl groups, with a few sulfate groups resulting from the sulfuric acid hydrolysis process [51]. The rejection of these membranes remains high, indicating that the presence of the CNCs did not significantly impact the structure of the polyamide skin layer. In the case of the 0.5 mg loading, a marked drop in water flux was observed (5.53 ± 0.31 LMH vs. 9.41 ± 0.45 LMH). This is likely due to a closely packed barrier layer of CNCs forming across the PES support, as observed in Figure 9. We believe the drop in flux at the 0.5 mg loading and the rise again at 0.75 mg loading to be a consequence of the unpredictability of the vacuum filtration fabrication method due to non-uniform packing and aggregation of the CNCs.

**Figure 11:**
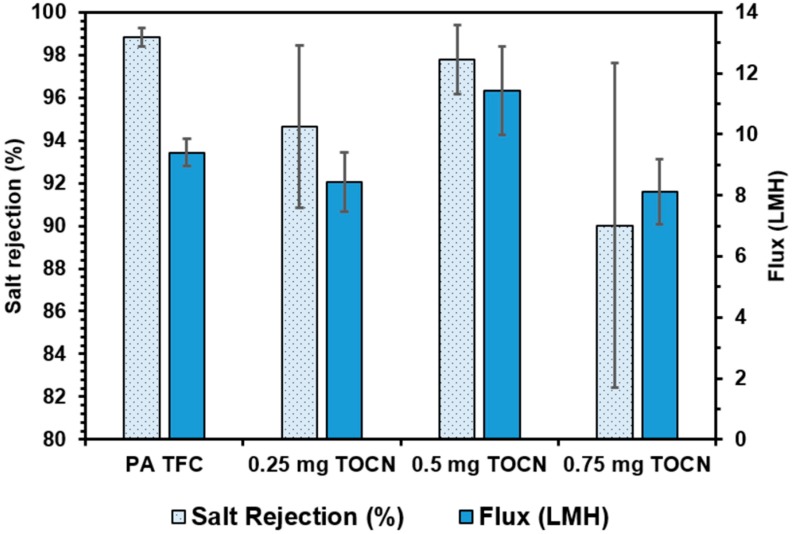
Flux and salt rejection results for TOCN containing TFN membranes fabricated using the vacuum filtration method.

The salt rejection and flux results for TFNs fabricated using TOCNs and the vacuum filtration method are displayed in Figure 11. Changes in the performance of the membranes are evident in both the rejection and flux values. The TOCN TFN membranes exhibited lower salt rejections at all loading levels: 94.6 ± 3.8% at 0.25 mg, 97.8 ± 1.6% at 0.5 mg, and 90.0 ± 7.6% at 0.75 mg, compared to 98.8 ± 0.4% in the plain PA membranes. The TOCN aggregates likely caused disruptions during interfacial polymerization, allowing larger network voids to form within the polyamide layer, and allowing increased salt transport through the membrane. No clear trend was observed for water flux as a function of TOCN loading level, with slight decreases observed at 0.25 mg and 0.75 mg, and a slight increase to 11.44 ± 1.61 LMH for the 0.5 mg loading compared to 9.41 ± 0.45 LMH for the control membrane. Thus, it appears that the vacuum deposited TOCNs are capable of affecting both the flux and salt rejection in the TFN membranes, but not in a consistent fashion. It is clear based on the results for both CNC and TOCN membranes fabricated using vacuum filtration that the method does not produce membranes in which the nanocrystals are uniformly and consistently dispersed throughout the polyamide layer.

#### 3.4.2. Dispersion Fabrication Method

**Figure 12:**
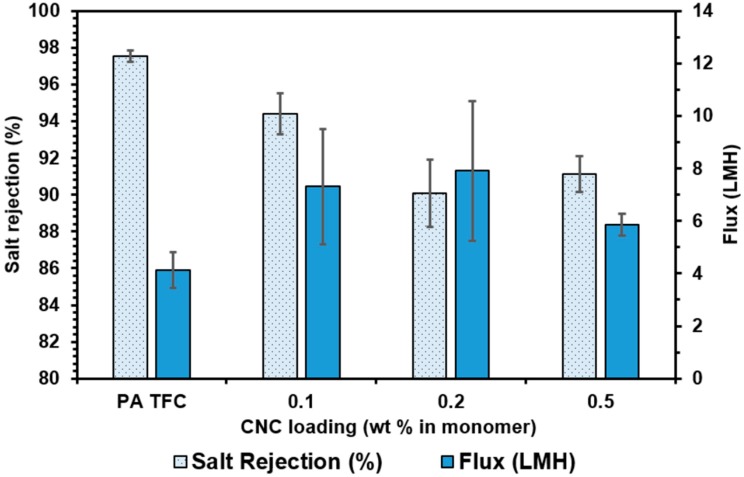
Flux and salt rejection results for CNC containing TFN membranes fabricated using the monomer dispersion method.

The permeation and salt rejection results for CNC-based membranes fabricated via the dispersion method are depicted in Figure 12. A marked increase in flux occurs at a 0.1 wt% loading level (7.3 ± 1.1 LMH vs. 4.13 ± 0.68 LMH for the control polyamide membrane). As the loading level is increased to 0.2 wt% a further increase is observed, followed by a slight decrease at 0.5 wt%. The increased fluxes suggest adequate dispersion of the CNCs in the polyamide layer and the formation of voids at the CNC/polymer interface that lead to more rapid water transport, as depicted in Figure 2. The increase in water flux is accompanied by a decrease in salt rejection to 95% at 0.1 wt% and ~91% at 0.2 wt% and 0.5 wt%. This behavior suggests that the voids formed at the CNC/polymer interface are not small enough to effectively prevent salt transport. This is likely due to the strength of the interactions at the nanoparticle-polymer interface. The CNCs possess surface hydroxyl functional groups that are capable of forming hydrogen bonds with the polyamide matrix, but will not interact as strongly as the surface carboxyl groups on the TOCNs. It should be noted that the PA control membranes reported here exhibit different RO performance than those presented in the preceding section (Figure 10 and Figure 11.) We believe that this is due to the inclusion of SDBS surfactant in the MPD solution during interfacial polymerization in the vacuum filtration method. Membranes made with the dispersion method did not have SDBS added to the MPD solution. However, it is clear that adding SDBS to the MPD solution improves the water flux in the membranes significantly, so this will be examined further in the future studies.

**Figure 13:**
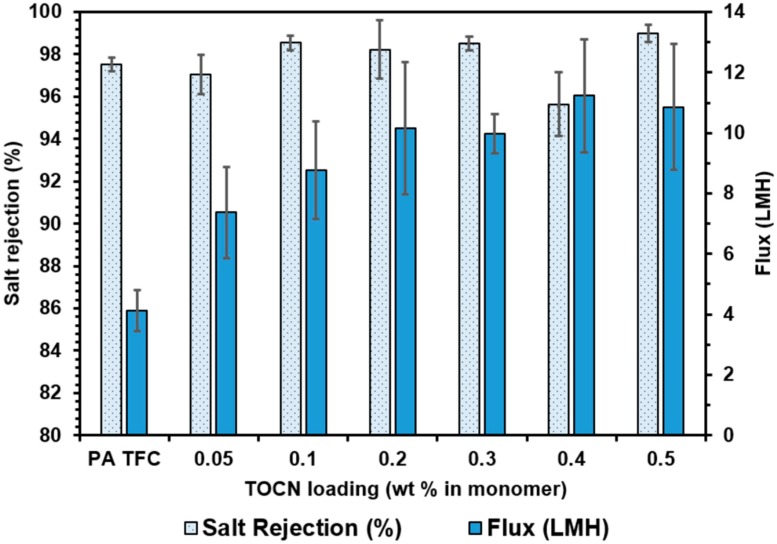
Flux and salt rejection results for TOCN containing TFN membranes fabricated using the monomer dispersion method.

The permeation and salt rejection data for TFN membranes fabricated via the monomer dispersion method and TOCNs are depicted in Figure 13. A clear trend of increasing water flux with increasing TOCN loading is observed. At the lowest loading level (0.05 wt% TOCN in monomer), a marked increase in flux was observed compared to the control TFC membrane (7.36 ± 1.51 LMH vs. 4.13 ± 0.68 LMH, respectively). The flux increases with TOCN loading level up to 0.5 wt% TOCN in the monomer solution before appearing to level off. At the highest loading level (0.5 wt%), the average flux was over two times higher than the control membrane (10.86 ± 2.08 LMH vs. 4.13 ± 0.68 LMH). The increasing flux is evidence that the monomer dispersion method is effective at achieving uniform dispersion of nanocrystals throughout the polyamide thin film. As the polymer layer forms during interfacial polymerization, the presence of the nanocrystals results in the formation of nanoscale voids that allow rapid water transport at the TOCN/polymer interface. Rejection values remained high at all loading levels, with the highest rejection observed for the 0.5 wt% loading level (98.98 ± 0.41%). The high salt rejection values indicate that there is a stronger interaction between the TOCN surface and the polyamide matrix than between the CNC and the polyamide matrix due to the increased hydrogen bonding ability of the carboxyl groups. The strengthened interactions lead to narrower channels at the TOCN/polymer interface that prevent salt transport. The carboxyl groups are also larger than the hydroxymethyl groups, potentially leading to an increased steric hindrance to salt transport.

Some variability in membrane fabrication and performance was still observed for the TOCN membranes produced via the dispersion method, as demonstrated by the relatively large error bars in Figure 13. This is especially evident at the 0.4 wt% loading level, where a marked drop in rejection was observed. This anomalous decrease is attributed to variations in the IP reaction conditions or incomplete dispersion of TOCNs in the monomer solution.

## 4. Discussion

We hypothesize that transport in the interfacial region between the nanocrystals and the polymer matrix is primarily responsible for the increased flux and high salt rejection in the nanocomposite membranes reported here. The presence of high aspect ratio nanoparticles incorporated into the polyamide matrix during interfacial polymerization results in the creation of new transport pathways through which water molecules can travel more rapidly than in the bulk polymer matrix, as depicted in Figure 2. However, the strength of the nanocrystal/polymer interactions is important in determining the size, and selectivity, of these transport pathways. Increased hydrogen-bonding between the TEMPO-oxidized CNCs and the polyamide matrix result in nanochannels that are small enough to sterically reject salt ions, leading to high salt rejection at all loading levels tested in this study. It is possible that better flux and rejection values may be obtained at higher TOCN loadings, although there is likely a point of diminishing returns at high loading levels due to TOCN aggregation or wholesale disruption of the polyamide matrix. In addition, the inclusion of other surface functionalities could allow the tuning of particle/polymer interactions to allow further control over water flux and salt rejection. Further study is necessary to optimize this membrane system.

## 5. Conclusions

Novel thin-film nanocomposite membranes containing cellulose nanocrystals have been fabricated using a vacuum filtration method previously established in our group as well as a new method involving dispersion of the nanocrystals in a monomer solution. The water flux and salt rejection performance of these membranes was evaluated using a reverse osmosis test apparatus and compared to control polyamide TFC membranes. The TFN membranes produced via the vacuum filtration method exhibited inconsistent results with CNC-based membranes exhibiting high salt rejection but some evidence of decreased water flux. The TOCN membranes fabrication via vacuum filtration exhibited little change in water flux, but decreased salt rejection. The AFM imaging of the vacuum deposited CNCs and TOCNs prior to interfacial polymerization revealed a closely packed layer of nanocrystals oriented predominantly in the plane of the PES support membrane, along with occasional large aggregates. This pre-polymerization structure leads to poor nanocrystal dispersion in the membrane, potential for decreased flux due to the tightly packed nanocrystal layer, and potential for decreased salt rejection due to disruption of the polyamide layer by large aggregates. The monomer dispersion method resulted in more consistent TFN formation for both CNCs and TOCNs. The well-dispersed nanocrystals cause nanoscale defects to form during the interfacial polymerization, which introduce new rapid water transport pathways at the nanocrystal/polymer interface. In the case of the CNCs, the increased flux is accompanied by decreased salt rejection, as the interfacial transport pathways are too large to effectively prevent salt transport. In contrast, the TOCN-based membranes exhibited increased water flus while maintaining high salt rejection values. Improved polymer/nanocrystal interactions, attributed primarily to increased hydrogen bonding between surface carboxyl groups and the polyamide matrix result in interfacial nanochannels that allow rapid water transport while preventing ion transport. To our knowledge, this is the first example of rapid molecular transport due to the inclusion of non-porous high aspect ratio particles in a polymer membrane. This work suggests that control of particle/polymer interactions in nanocomposite membranes containing high aspect ratio nanoparticles can be used to tune molecular transport and selectivity.

## Figures and Tables

**Table 1:** Polyamide skin layer thickness determined from TEM imaging of samples.

Membrane	Polyamide Layer Thickness (nm)
Polyamide control (FT-30)	360 ± 70
TFN 0.5 mg CNCs	310 ± 40
TFN 0.5 mg TOCNs	365 ± 70

**Figure A1:**
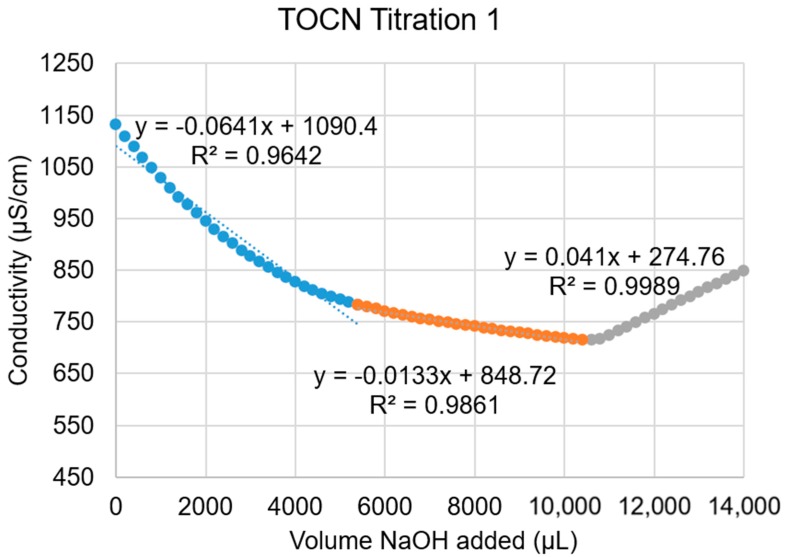
Conductivity vs. volume NaOH added for the first TOCN titration.

**Figure A2:**
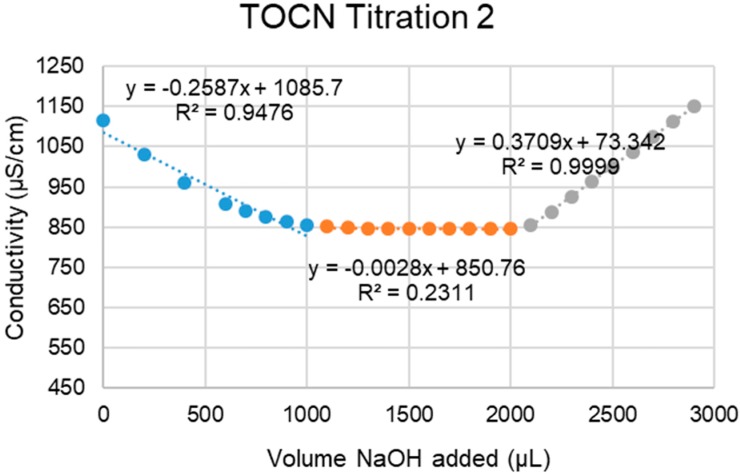
Conductivity vs. volume NaOH added for the second TOCN titration.

**Figure A3:**
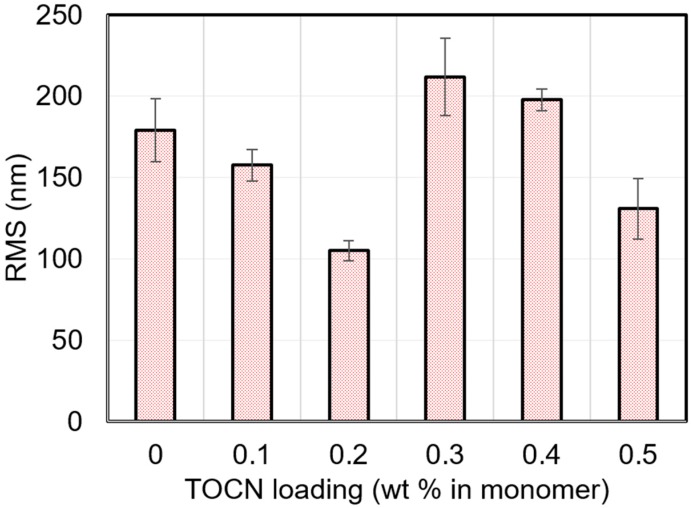
RMS roughness data obtained from AFM scans of TFN membranes with various TOCN loading amounts.

**Figure A4:**
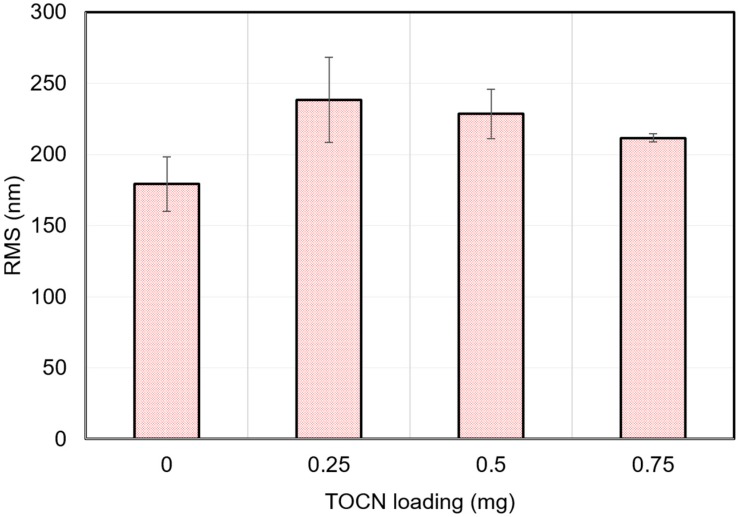
RMS roughness data from AFM scans of membranes fabricated with TOCNs via the vacuum filtration method.

**Figure A5:**
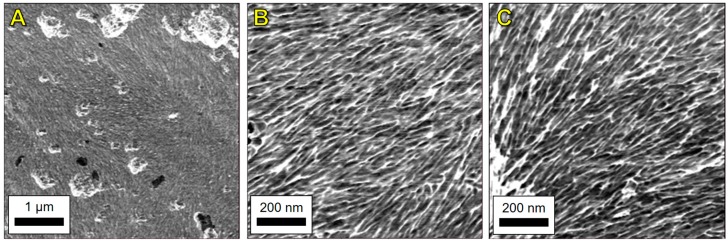
AFM phase retrace images of (**A**) 0.5 mg TOCN at 5 µm scan size, (**B**) 0.5 mg TOCN at 1 µm scan size, and (**C**) 0.75 mg TOCN at 1 µm scan size. These phase images correspond with the height retrace images presented in Figure 7, Figure 8a,b, respectively.

**Figure A6:**
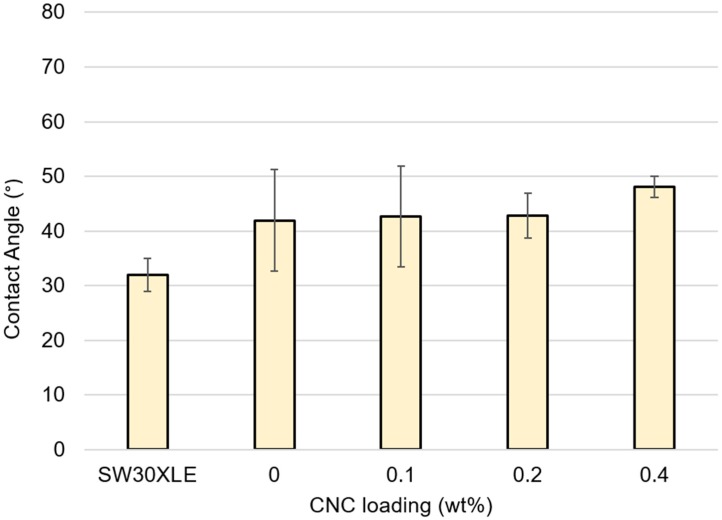
Contact angle measurements for TFN membranes with loading levels of unmodified CNC between 0 and 0.4 wt%. For comparison, measurements from a DOW SW30XLE industrial membrane are included.

**Figure A7:**
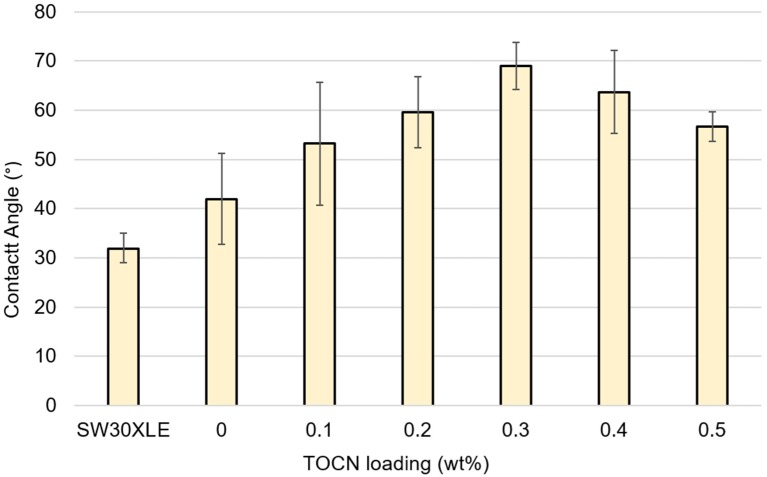
Contact angle measurements for TFN membranes with TOCN loading levels from 0 to 0.5 wt%. For comparison, measurements from a DOW SW30XLE industrial membrane are included.

## Appendix A

### A.1. Thin-Film Nanocomposite Performance

**Table A1:** Comparison of TFN membrane performances in recent literature. Note: All studies were performed using a feed solution of 2000 ppm NaCl, with the exception of the study with no nanoparticles, which used 1500 ppm NaCl.

Nanomaterials	Applied Pressure (psi)	Water Flux (L/m^2^·h)	Rejection (%)	Pure Water Permeability (L/m^2^·h·bar)
None [52]	217	42	99.8	2.81
SW-CNTs [8]	350	36.2	>98	1.5
Ag [13]	300	69.4	93.6	3.36
Silica [14]	250	15	95.1	0.87
MIL-101 (Cr) [9]	232	52.8	99.1	3.3
TiO_2_ [17]	300	65	97	3.14

### A.2. TEMPO Oxidation of Cellulose Nanocrystals

As-received cellulose nanocrystals were oxidized via the following procedure: 100 g of 11.8 wt% CNCs were added with 674 mL of DI water, 24 mL were used to clean weight dish and added by transfer pipette while 650 mL were added by graduated cylinder; 0.3255 mg TEMPO free radical and 3.5635 g NaBr were added to a vessel followed by 200 mL DI water (Sybron Barnstead P/N16508) by graduated cylinder. NaBr solution was added to cellulose nanocrystal solution. Approximately 54.7 mL of NaClO solution was measured by graduated cylinder and slowly added to CNC solution in large Büchner flask while it was stirring. Two-hundred mL of NaOH solution was produced using 4.0665 g of NaOH and a difference in volume of water making 0.5 M NaOH solution. Using NaOH solution, pH was adjusted to 10, measured with a pH probe (Fisher Scientific Orion Star A215), and kept constant for three hours adding by NaOH dropwise. After 3 h, 40 mL of ethanol was added by graduated cylinder after pH was kept constant for 3 h. The TOCN solutions were placed in dialysis tubing, and submerged in DI water. The water was changed several times over the next few days. When the resistivity of the TEMPO CNC bath was 16.53 MΩ·cm. The CNCs were found be in a concentration of ~5.5 mg/mL.

### A.3. Conductometric Titration of TOCNs

In order to determine the surface –COOH group concentration on the TOCNs, a conductometric titration was performed using a Fisher Scientific Orion Star A215 conductivity probe; 9.1 mL of the 5.5 mg/mL TOCN stock solution was combined with 0.2 mL of 0.5 M hydrochloric acid, 5 mL of a 0.01 M potassium chloride solution, and 15.7 mL of deionized water. A solution of 0.05 M NaOH was added dropwise to the TOCN-acid solution, and the volume of NaOH solution added (µL) and conductivity readings (µS/cm) were recorded. When the conductivity returned to near its original value at the beginning of the titration, the process was stopped. The procedure was repeated, and the resulting conductivities were plotted against the volume of NaOH solution added to generate Figure A1 and Figure A2:

Equations were generated to fit each region of the curves, as indicated in Figure A1 and Figure A2 by the blue, orange, and gray colored regions. Intersection points were found between the lines to establish the volume at which the orange region began (this region represents the volume of NaOH needed to titrate the –COOH groups present on the TOCNs). This volume was then used in Equation (A1) to calculate the approximate –COOH end group density on the TOCNs:

(A1) $$\frac{C_{NaOH}V_{NaOH}}{m_{TOCN}},$$

where $C_{NaOH}$ is the concentration of the NaOH solution (M), $V_{NaOH}$ is the volume of NaOH needed to titrate the –COOH groups present in the TOCN solution (orange regions on the curves in Figure 2 and Figure 3), and $m_{TOCN}$ is the total mass of TOCNs present in the solution in grams. The values for TOCN surface density were determined to be 1.16 mmol/g cellulose for both titrations, which is comparable to literature results [39,40,41].

### A.4. TEM Characterization of TOCNs

Previously developed TEM techniques [45] were used to characterize the TOCNs used in this study. The TOCNs were diluted to 0.1 mg/mL then added a drop to a transmission electron microscopy (TEM) grid (Pelco^®^ 200 Mesh copper coated silicon monoxide) by transfer pipette then adding 1 drop of Nanovan^©^ stain for thirty seconds and sheeting off excess with a water dunk. The sample was then allowed to dry at room temperature, and characterized using a JEOL 2100 TEM. Images were taken slightly out of focus to enhance contrast. Length and width of the TOCNs were determined to be approximately 101 ± 8 nm and 7 ± 1 nm, respectively, by processing the images presented in Figure 1 with Fiji [38].

### A.5. AFM Root Mean Square Roughness

The RMS roughness data were collected from AFM images of each membrane fabricated via the dispersion method at each loading level. The results are displayed in Figure A3.

These data are provided in numerical form in Table A2.

**Table A2:** Numerical RMS roughness values for membranes fabricated with TOCNs via the dispersion method.

TOCN Loading (wt % in Monomer)	RMS (nm)
0	179.2 ± 19.3
0.1	157.6 ± 9.7
0.2	105.1 ± 6.3
0.3	211.8 ± 23.9
0.4	197.8 ± 6.9
0.5	130.8 ± 18.6

The RMS roughness data were also obtained for membranes fabricated via the vacuum filtration method. Figure A4 shows these results.

Table A3 displays these as numerical results.

**Table A3:** Numerical RMS roughness values for membranes fabricated with TOCNs via the dispersion method.

TOCN Loading (mg)	RMS (nm)
0	179.2 ± 19.3
0.25	240 ± 30
0.5	228 ± 17
0.75	212 ± 3

### A.6. AFM Phase Images

Figure A5 displays the phase retrace images of the scans in Figure 7 and Figure 8.

### A.7. Contact Angle Goniometry

Contact angle goniometry was used to evaluate the surface hydrophobicity of selected membranes fabricated during this study. A DOW SW30XLE commercial TFC membrane was also analyzed for comparison purposes. One square centimeter areas were cut from the desired membrane and fastened to glass slides. Deionized water droplets approximately 25 µL in volume were dropped onto each surface, and images were taken via the goniometer (CAM 200, KSV Instruments, Ltd., Helsinki, Finland) after one minute of droplet exposure. Measurements were obtained from at least three locations across each membrane. The left and right contact angles were averaged to obtain the final values. The results of these measurements are presented in Figure A6 and Figure A7.
